# Beet root carbon dots cellulose sulfate film as a novel naked eye pH sensor for chromium and bacterial detection in tomatoes 

**DOI:** 10.1038/s41598-025-15663-9

**Published:** 2025-08-18

**Authors:** Hebat-Allah S. Tohamy

**Affiliations:** Cellulose and Paper Department, National Research Centre, 33 El Bohouth Str, P.O. 12622, Dokki Giza, Egypt

**Keywords:** Chromium sensor, Bacterial sensor, Food packaging, Biosensors, Color-sensing, PH-sensing, Fluorescent sensor, Beet root carbon dots, Environmental sciences, Chemistry, Materials science, Nanoscience and technology

## Abstract

This study presents the development and characterization of a novel nitrogen doped carbon dots cellulose sulfate-carboxymethyl cellulose composite film (N–CDs-CS-CMC) for multifunctional applications in tomato packaging and sensing. Density functional theory (DFT) calculations revealed a significant enhancement in polarity (29.19 Debye) and a reduced energy gap (0.019 eV) for the composite compared to CS-CMC, indicating improved molecular interactions and enhanced charge transfer. Scanning electron microscopy (SEM) showed a surface with smaller, more uniform pores (11.93–25.45 μm), increasing surface area and enhancing sensing capabilities. The N–CDs-CS-CMC film exhibited potent antimicrobial activity against *Escherichia coli*, *Staphylococcus aureus*, and *Candida albicans*, with inhibition zones of 20, 22, and 19 mm, respectively, and demonstrated distinct fluorescence patterns upon bacterial interaction, enabling pathogen-specific detection. Incorporating beetroot (BR) within N–CDs preparation method rendered the film pH-responsive, showing color changes from brown/reddish in alkaline to yellow in acidic environments. Tomatoes wrapped with the N–CDs-CS-CMC film exhibited a 10-day shelf life, compared to 4 days for CS-CMC. Furthermore, the film underwent rapid decolorization to yellow and increased tomato translucency upon exposure to chromium, indicating its potential for heavy metal detection. These results demonstrate the N–CDs-CS-CMC film’s efficacy as a multifunctional material for enhanced food packaging, integrating antimicrobial properties, bacterial and chromium sensing, and pH monitoring for improved food safety and quality.

## Introduction

Ensuring food safety is a paramount global concern, particularly regarding the presence of heavy metals and pathogenic bacteria in processed and fresh produce^1,2^. Tomatoes, a widely consumed vegetable, are susceptible to contamination by chromium (Cr) and bacterial pathogens, posing significant risks to human health^3,4^. A particularly concerning source of chromium contamination stems from the leaching of Cr from the interior linings of canned food containers. This process can result in elevated Cr levels in canned tomatoes and tomato-based products, leading to potential long-term health hazards ^4–6^. Conventional techniques for detecting chromium and bacteria in food samples, such as atomic absorption spectroscopy and microbial culturing, are often laborious, require skilled personnel, and are not suitable for on-site analysis. This necessitates the development of novel sensing platforms that offer simplicity, sensitivity, and real-time detection capabilities ^7–11^. Tomatoes are a staple food worldwide, prized for their nutritional value and versatility^12,13^. However, their vulnerability to contamination by heavy metals, specifically chromium, and bacterial pathogens raises serious concerns for consumer safety^14,15^. Therefore, the development of efficient and reliable methods for detecting these contaminants is crucial for maintaining the quality and safety of tomato products. To maintain product quality and detect food spoilage, industries utilize various packaging techniques, including smart and active packaging ^16,17^.

In line with the growing emphasis on sustainable and eco-friendly technologies, this research explores the utilization of beet root (BR), a readily available and natural resource, for the synthesis of carbon dots (CDs). While organic dyes and metal-based CDs possess fluorescence, their use is limited by toxicity and instability ^18–22^. This study capitalizes on the inherent optical properties of beet root, specifically its betalain pigments, to synthesize CDs. BR naturally contains betalain pigments. Betalains, a group of water-soluble pigments biosynthesized by plants through the shikimate pathway from betalamic acid, serve as the chromophoric source. The immonium derivative of betalamic acid yields yellow pigments (betaxanthins), while its condensation product with cyclodihydroxyphenylalanine (cyclo-DOPA) produces purplish-red pigments (betacyanins) ^23^. These inherent color variations within betalains provide a basis for naked-eye colorimetric detection, directly correlating with changes in the chemical environment or the presence of target analytes. Consequently, the color of the synthesized CDs, derived from these BR, can be visually assessed, offering a simplified approach to rapid, on-site analysis. These BR-derived CDs offer a safer and more sustainable alternative, exhibiting fluorescence visible to the naked eye, thus eliminating the requirement for complex instrumentation.

The integration of these bio-derived CDs with cellulose-based polymers creates a biocompatible and biodegradable film, offering a green alternative for contaminant detection ^24–29^. As the most abundant renewable resource, cellulose offers valuable derivative materials when their properties are tailored for specific uses ^30–32^. Cellulose ethers, a group of semi-synthetic polymers, are created by chemically reacting the hydroxyl groups on the D-glucopyranose units of cellulose, which are connected by β(1→4) glycosidic linkages ^33–38^. In food packaging, cellulose derivatives have proven versatile, effectively retarding the migration of food components like moisture, while also serving as carriers for nutrients such as antimicrobial agents. Commercially available sodium carboxymethylcellulose (CMC)-based edible coatings for fruit. Therefore, the utilization of cellulose derivatives in food packaging represents a promising avenue for enhancing food preservation and quality through their multifunctional capabilities ^34,39^. Due to its antimicrobial, biocompatibility and biodegradability, water-soluble cellulose sulfate (CS) is a preferred material for various food packaging applications ^40^. CS is derived from cellulose through the substitution of hydroxyl groups (OH) with sulfate groups (SO_3_H), either partially or completely ^40,41^. However, the inherent brittleness of CS films limits their practical application ^42^. To address this limitation and to enhance the film’s flexibility and control the release of active compounds, we incorporated carboxymethylcellulose (CMC) as a plasticizer ^43,44^. CMC, a compatible cellulose derivative, is known to improve film-forming properties and modulate release kinetics. Therefore, this study explores the fabrication and characterization of a CS/CMC composite film, aiming to develop a material with improved properties, including tailored CDs release capabilities. Sugarcane bagasse (SB), a readily available agrowaste, was utilized as the starting material for the production of both CMC and CS. Initially, the SB was thoroughly cleaned and milled to a fine powder. To enhance cellulose accessibility, the powder underwent alkaline pretreatment, effectively removing lignin and hemicellulose, followed by bleaching to isolate pure cellulose. The isolated cellulose was then subjected to carboxymethylation, involving activation with an alkaline solution and reaction with monochloroacetic acid, ultimately yielding CMC, which was subsequently purified and dried ^37,44^. For the synthesis of CS, the cellulose derived from sugarcane bagasse was again employed. The cellulose underwent a sulfation reaction, where it was activated and reacted with a sulfating agent under controlled conditions ^45^. Utilizing this abundant agricultural waste stream aligns with principles of a circular economy, transforming a low-value byproduct into valuable industrial materials. The ability to detect chromium and bacteria through naked-eye observation offers a significant advantage in terms of simplicity and accessibility, especially for on-site applications. The colorimetric response of the film, coupled with its fluorescence properties, provides a straightforward and rapid method for assessing the safety of tomato samples.

## Materials and methods

### Materials

Beet root (BR), sourced from a local Egyptian market, was employed to synthesize nitrogen doped carbon dots (N–CDs). The urea and sodium hydroxide were purchased from Sigma-Aldrich. All materials were used as received without further purification.

### Preparation of nitrogen doped carbon Dots (N–CDs)

Nitrogen-doped carbon dots (N–CDs) were produced from beet root (BR) through a multi-step process. This involved creating a uniform mixture of 4 g BR, 9.33 g NaOH, 9.33 g urea, and 100 mL of water, followed by sequential treatments: freezing, sonication, and microwave irradiation at 700 W for 7 min.

### Preparation of cellulose

Initially, 150 g of SB underwent prehydrolysis in an autoclave with dilute hydrochloric acid at 120 °C for 2 h. Following this, the pretreated material was subjected to an alkaline treatment using 20 g of sodium hydroxide dissolved in 300 mL of water, heated to 170 °C for 2 h, yielding a brownish, fibrous pulp. To remove lignin, 80 g of the pulp was bleached with a 3% solution of HClO_2_ (2.4 g in 4750 mL of water) in an acetic acid environment at 80 °C for 2 h. The pH was maintained between 1 and 3 by controlled additions of acetic acid, resulting in purified α-cellulose ^37,44,46^.

### Preparation of cellulose sulfate (CS)

Deep eutectic solvent was synthesized by mixing sulfamic acid and urea (1:2 molar ratios) and heating at 80 °C with magnetic stirring until clear. Subsequently, cellulose (10:1 Sulfamic acid: cellulose molar ratio) was added and homogenized with a glass rod. The reaction mixture was then heated to 150 °C and held for 30 min. After a brief cooling period, the reaction was terminated by water addition. The cellulose sulfate (CS) was recovered through filtration and washed to neutrality, then stored at 4 °C ^45^.

### Preparation of carboxymethyl cellulose (CMC)

Cellulose (15 g) was combined with a 30% sodium hydroxide solution, and 18 g of monochloroacetic acid was added. This mixture was then heated via microwave irradiation until complete dissolution occurred. Carboxymethyl cellulose (CMC) was subsequently precipitated using 70% ethanol, isolated by filtration, and dried in an oven ^16,46^.

### Preparation of nitrogen doped carbon dots-cellulose sulfate-carboxymethyl cellulose film (N–CDs-CS-CMC)

A composite film composed of N–CDs, CS, and CM was prepared via solution casting in a Teflon plate. Specifically, 0.5 g of CS and 0.5 g of CMC were dissolved in 50 mL of deionized water. The N–CDs-containing film was fabricated by incorporating 15% (wt/wt) into the CS-CMC solution and denoted as N–CDs-CS-CMC. A control film (blank) was prepared following the same procedure but without the addition of N–CDs and denoted as CS-CMC.

### Nitrogen doped carbon dots-cellulose sulfate-carboxymethyl cellulose film application to monitor and preserve chromium and bacteria in tomatoes

Fresh tomatoes, acquired from a local Cairo market in Egypt, were used in an experiment. In order to test the films’ ability to detect chromium, tomatoes were prepared to contain a chromium concentration of 1.85 mg/kg, a level consistent with reported contamination in canned tomatoes^4^. These polluted tomatoes were then used for the sensing experiments. Two tomatoes were individually enclosed within containers, each wrapped with a different film: one made of CS-CMC (non-colorimetric) and the other with N-CDs-CS-CMC (colorimetric). These containers were then stored at 25 °C for 10 days. Throughout this period, the color changes of the tomatoes were monitored and documented using a smartphone camera.

### Characterization

#### Evaluating the pH sensitivity of intelligent films

To assess the film’s pH sensitivity, we followed the protocol outlined by Tohamy et al. The film was exposed to acidic and alkaline buffer solutions for 30 s. A smartphone was used to document any visible color changes ^17^.

#### Morphological examination

Microstructural characterization was performed using a Quanta/250-FEG scanning electron microscope (SEM) (Thermo Fisher Scientific, Waltham, MA, USA).

#### Fluorescence microscope

Fluorescence measurements were conducted using a Jasco FP-6500 spectrofluorometer equipped with a 150-watt xenon arc lamp.

#### Fourier-transform infrared (FTIR) spectra

Fourier Transform Infrared (FTIR) spectroscopy was performed using a Mattson 5000 spectrometer (Unicam, United Kingdom). KBr pellet method was employed for sample preparation. The mean hydrogen bond strength (MHBS) was determined using Eq. (1):

1 $$\:\text{M}\text{H}\text{B}\text{S}=\frac{{A}_{OH}}{{A}_{CH}}$$

where A_OH_ and A_CH_ refer to the FTIR absorbance of the OH and CH peaks, respectively ^25,26,47^.

#### DFT calculations

Computational investigations were conducted using density functional theory (DFT), implemented within the Gaussian 09 W software. Geometry optimization was achieved through the Berny algorithm. This methodology allowed for the exploration of various parameters, including optimized molecular structures and their corresponding ground state energies. The total energy (E_T_), the energy of the highest occupied MO E_HOMO_, the energy of the lowest unoccupied MO E_LUMO_, the energy gap (E_g_), the dipole moment (µ), the absolute hardness (η), the absolute softness (σ), the chemical softness (S), and the additional electronic charge (ΔN_max_) were calculated ^11,48,49^.

2 $$\:{E}_{gap}=({E}_{LUMO}-{E}_{HOMO})$$

3 $$\:{\upeta\:}=\frac{({E}_{LUMO}+\:{E}_{HOMO})\:\:}{2}\:$$

4 $$\:{\upsigma\:}=\frac{1\:\:}{{\upeta\:}}$$

5 $$\:\text{S}=\frac{1\:\:}{2{\upeta\:}}$$

6 $$\:{\Delta\:}{N}_{max}=\frac{-\text{P}\text{i}\:\:}{{\upeta\:}}$$

#### Molecular Docking

The molecular docking of N–CDs-CS-CMC against *Escherichia coli* PDB (7AB5), *Staphylococcus aureus* PDB (4QLO), and *Candida Albicans* PDB (1YR1) as a receptor. The protein complex was fabricated using standard bond length, with the Gaussian 09 W and detected by discovery Studio Client (version 4.2). The generated poses were then evaluated based on their calculated binding affinity. The pose with the lowest bond length was selected as the most probable binding conformation for further analysis.

## Results and discussion

### DFT calculations

To elucidate the stability and electronic properties of CS-CMC, N–CDs, and their composite (N–CDs-CS-CMC), density functional theory (DFT) calculations were employed. The analysis of Table 1 yielded significant insights into the molecular interactions and electronic structure of these materials. The calculated µ serves as a metric for the overall polarity of a molecule or composite. The observed substantial increase in µ for the N–CDs-CS-CMC composite (29.19 Debye) compared to CS-CMC (14.58 Debye) indicates a significant enhancement in polarity. This increase can be attributed to the incorporation of N–CDs, which introduce a higher density of electronegative nitrogen (N) and oxygen (O) atoms into the CS-CMC matrix ^16,17^. These heteroatoms create a more uneven distribution of electron density, leading to a larger dipole moment. This increased polarity is vital for adhesion and compatibility with the tomato surface, ensuring efficient packaging and preventing delamination. Furthermore, higher polarity can facilitate the interaction and binding of compounds emitted by tomatoes during ripening or spoilage to the film’s surface, which is essential for sensing applications. Additionally, increased polarity might influence the film’s sensitivity to moisture, a critical factor in food packaging.

The calculated E_g_ provides insights into the electronic properties and potential sensing capabilities of the film. The observed lowest E_g_ for the N–CDs-CS-CMC composite (0.019 eV) suggests a strong chemical interaction between the components and indicates enhanced charge transfer within the composite, which is essential for developing sensitive and responsive sensors. A lower E_g_ signifies higher chemical reactivity, which is crucial for the film to interact with and detect target analytes ^11,49^. The introduction of N–CDs creates electronic states within the CS-CMC matrix, allowing for more efficient electron transfer during sensing events. The strong chemical interactions implied by the low E_g_, likely through covalent or electrostatic bonding, ensure the structural integrity and stability of the film, which is vital for long-term packaging and sensing applications. Finally, the lower E_T_ of N–CDs-CS-CM (– 3152.50 au) suggests that the formation of the N–CDs-CS-CM composite is releasing energy during its formation. This energy release contributes to enhanced stability and resistance to bond breakage ^48^.

**Table 1:** The quantum chemical parameters of CS-CMC, N–CDs, and N–CDs-CS-CMC.

DFT B3LYP/6–31G (d)	CS-CMC	*N*–CDs	*N*–CDs-CS-CMC
E_LUMO_ (eV)	– 0.136	0.077	– 0.121
E_HOMO_ (eV)	– 0.167	– 0.373	– 0.141
E_g_ (eV)	0.030	0.450	0.019
E_T_ (au)	– 3112.37	– 244.29	– 3152.50
µ (Debye)	14.58	2.98	29.19
ɳ (eV)	– 0.151	0.225	– 0.131
σ (eV)	– 6.595	4.436	– 7.620

### Fourier transform infrared spectroscopy (FTIR) spectra

**Fig. 1:**
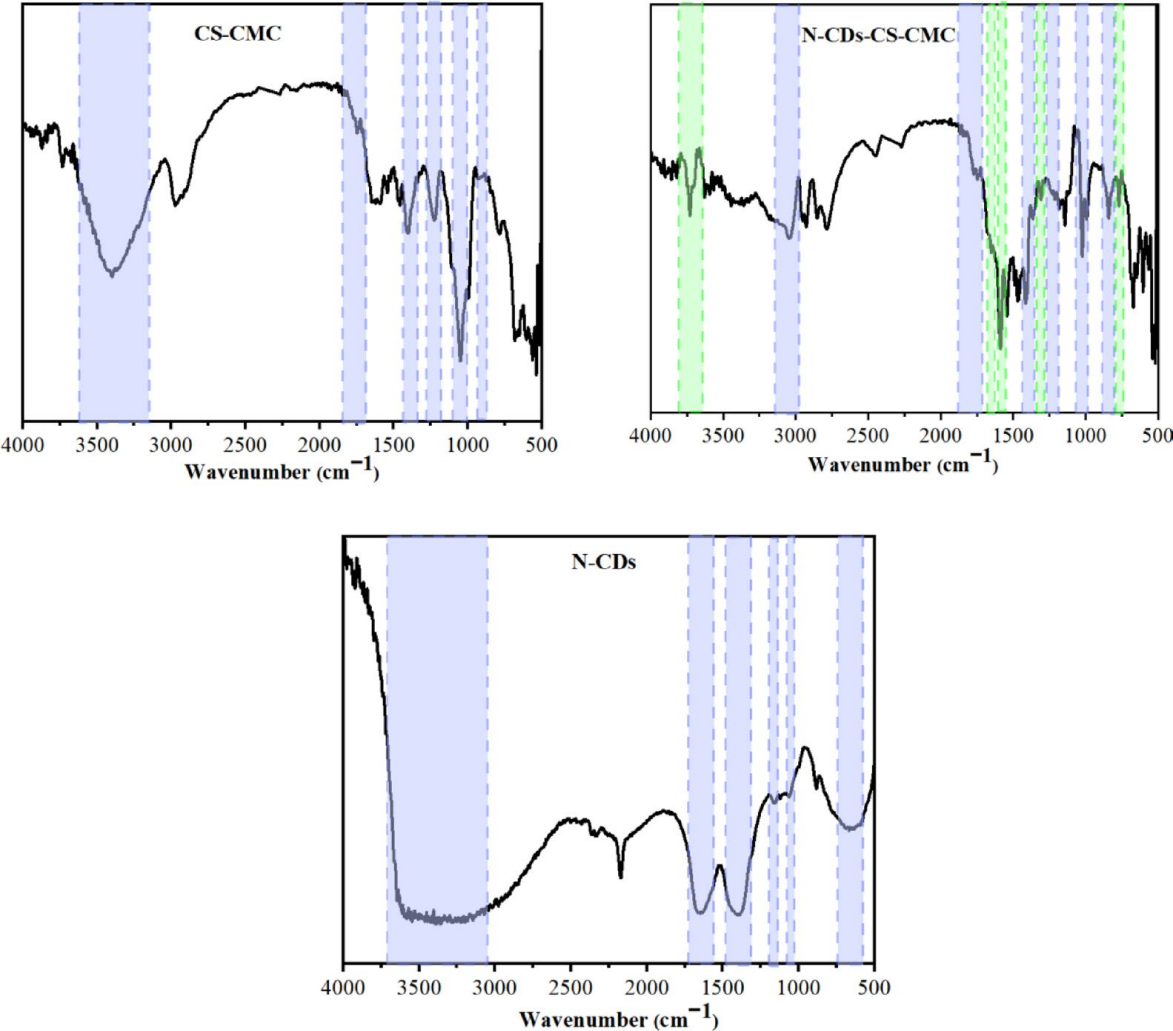
FTIR spectra of CS-CMC and N–CDs-CS-CMC and N–CDs. The green shadowed regions are the additional peaks which are appeared in N–CDs-CS-CMC compared to CS-CMC.

The N–CDs show peaks at 3535 cm^– 1^ (O–H/N–H), 1662 cm^– 1^ (C = O), 1404 cm^– 1^ (C–O bending), 1153 cm^– 1^ (C–O = C), 1062 cm^– 1^ (C–O–C), and 883 cm^– 1^ (C–N) ^25,26^. The CS-CMC showed peaks at 3405 cm^– 1^ (O–H), 1747 cm^– 1^ (C = O), 1401 cm^– 1^ (C–O bending), 1228 cm^– 1^ (S = O), 1052 cm^– 1^ (C–O–C pyranose ring), and 997 cm^– 1^ (C–O–S) ^45,46^. The N–CDs-CS-CMC showed the same peaks with additional peaks at 3465 cm^– 1^ (N–H), 1656 cm^– 1^ (amide I), 1587 cm^– 1^ (amide II), 1309 cm^– 1^ (O–C = O), and 840 cm^– 1^ (C–N) which prove the incorporation of N–CDs within N–CDs-CS-CMC (Fig. 1) ^11,48^. The additional peaks of N–CDs (e.g., N–H at 3465 cm⁻¹, amide I at 1656 cm⁻¹, amide II at 1587 cm⁻¹) in the N–CDs-CS-CMC film compared to CS-CMC; and the progressive shifts in the O–H peak (e.g., from 3405 cm⁻¹ in CS-CMC shifting towards 3046 cm⁻¹ in the N–CDs-CS-CMC film) indicating a greater extent of hydrogen bonding.

Regarding the mechanism of action between CS, CMC, and the N–CDs, a more profound interpretation is necessary. The primary interaction is strongly indicated by hydrogen bonding. The observed shift of the O–H stretching peak from 3405 cm⁻¹ in the CS-CMC film to a lower wavenumber of 3046 cm⁻¹ in the N–CDs-CS-CMC composite directly signifies an increase in hydrogen bond strength. This occurs because the N–CDs, with their abundant surface hydroxyl, carboxyl, and amino groups (due to nitrogen doping), serve as excellent hydrogen bond donors and acceptors. These N–CDs functional groups form extensive intermolecular hydrogen bond networks with the numerous hydroxyl and carboxyl groups present on the CS and CMC polymer chains, effectively “cross-linking” the components at a molecular level and contributing to the composite’s structural integrity. This is further substantiated by the calculated Mean Hydrogen Bonding Strength (MHBS) values, which showed an increase from 0.97 for CS-CMC to 1.00 for N–CDs-CS-CMC ^33,50^ Beyond hydrogen bonding, other non-covalent interactions may also contribute to the stable integration of N–CDs. Depending on the surface charge of the N–CDs (e.g., if amino groups are protonated, leading to a positive charge), electrostatic interactions could play a role with the negatively charged sulfate groups of CS and the carboxylate groups of CMC. While not directly observed by FTIR, physical entanglement is also an inherent part of nanoparticle-polymer composites, where the small N–CDs become physically entrapped within the forming polymer network. These combined molecular interactions ensure the robust and homogeneous dispersion of N–CDs within the CS-CMC matrix, which is critical for the composite film’s enhanced properties and overall performance in sensing applications.

The FTIR spectrum of CS-CMC and N–CDs-CS-CMC are differed as evidenced by the increased relative absorbance (RA) of the characteristic absorbance peaks of O–H (i.e. 0.97 and 1.00) and C = O (i.e. 1.00 and 1.06) for S CS-CMC and N–CDs-CS-CMC, respectively. The oxygenated groups are high in N–CDs-CS-CMC compared to the CS-CMC due to the presence of N–CDs.

### Morphological observations

**Fig. 2:**
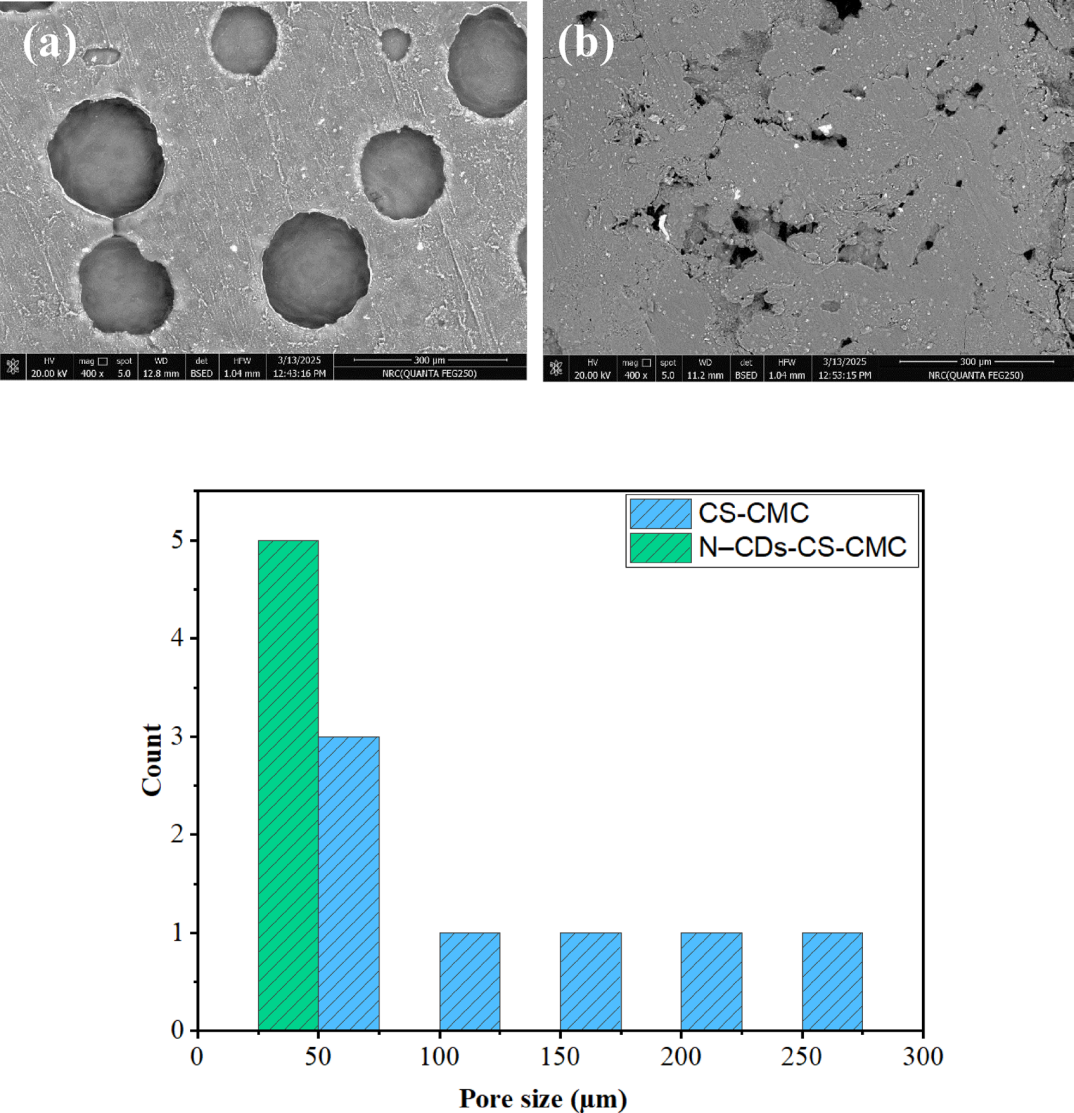
SEM analysis for (**a**) CS-CMC and (**b**) N–CDs-CS-CMC with the pore size distribution range.

The SEM analysis revealed distinct differences in the surface morphology of the CS-CMC and N–CDs-CS-CMC films, highlighting the superior potential of the N–CDs-CS-CMC film for advanced sensing applications, specifically for detecting bacteria and chromium in tomato environments. The CS-CMC film exhibited a surface characterized by large, circular pores, spanning a size range of 67.78 to 272.20 μm, rendering it less suitable for precise sensing (Fig. 2a). In contrast, the N–CDs-CS-CMC film displayed a surface with smaller, irregular pores, ranging from 11.93 to 25.45 μm, making it significantly more promising for sensitive and selective detection (Fig. 2b; Table 2). This observed difference in pore size and distribution is crucial for understanding the films’ potential, with the N–CDs-CS-CMC film demonstrating a clear advantage for capturing bacteria and facilitating the interaction of chromium ions with the sensing components.

The smaller, more numerous pores observed in the N–CDs-CS-CMC film contribute to a significantly higher surface area compared to the CS-CMC film. This increased surface area is advantageous for both bacterial and chromium sensing. For bacterial sensing, it enhances the contact points between the film and bacteria, potentially increasing capture efficiency. For chromium sensing, it provides more interaction sites for chromium ions, improving sensitivity. Furthermore, the pore morphology influences the diffusion and mass transport of bacterial metabolites and chromium ions through the film. The smaller pores in the N–CDs-CS-CMC film facilitate a more controlled diffusion, leading to a more sensitive and selective sensor response. Conversely, the larger pores in the CS-CMC film result in less controlled diffusion, compromising the precision of the sensor. Additionally, pore size impacts the film’s permeability to gases and moisture, which are critical factors in tomato packaging and sensing. The N–CDs-CS-CMC film’s smaller pores offer better control over gas permeability, creating a modified atmosphere around the tomato and extending its shelf life, while simultaneously allowing for controlled diffusion of bacterial metabolites. Moreover, the film’s surface morphology directly influences the sensor’s response and sensitivity. The enhanced interaction between the sensing elements (i.e. N–CDs) and the target analytes (bacteria and chromium ions), facilitated by the smaller pores in the N–CDs-CS-CMC film, leads to a stronger and more reliable signal. Thus, the SEM results strongly suggest that the N–CDs-CS-CMC film, with its favorable pore morphology, holds greater promise for developing effective packaging capable of simultaneous bacterial and chromium sensing compared to the CS-CMC film.

**Table 2:** Pore size for CS-CMC and N–CDs-CS-CMC.

Film	Pore size (µm)
CS-CMC	67.78–272.20
N–CDs-CS-CMC	11.93–25.45

### Antibacterial activity and molecular Docking study

**Fig. 3:**
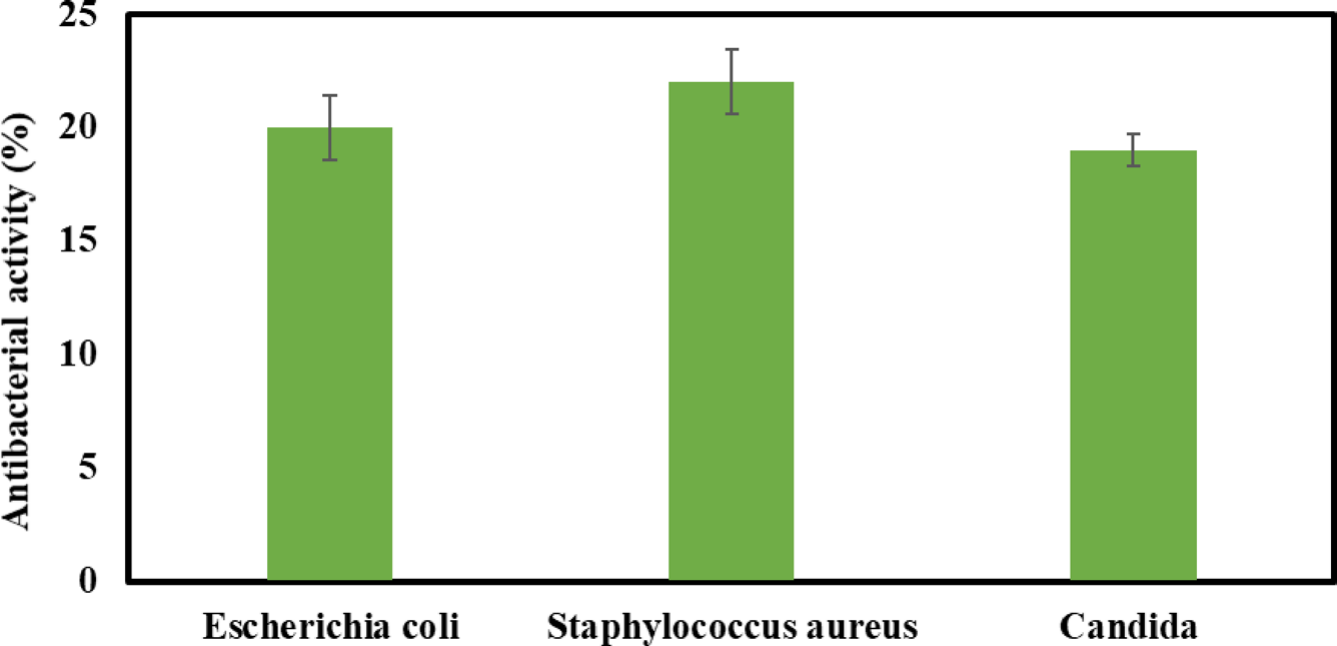
Inhibition zones with error bars for B2.

Post-harvest spoilage of tomatoes is significantly compromised by microbial contamination, with reports indicating *Escherichia coli*, *Staphylococcus aureus*, and *Candida* as primary spoilage agents^51–53^. These microbial agents underscore the necessity of stringent sanitation practices during cultivation, harvesting, processing, and storage to mitigate microbial load and prolong tomato shelf life. The findings revealed that CS-CMC (denoted as B1) showed no antibacterial activity against the three species. On contract the N–CDs-CS-CMC (denoted as B2) exhibited antibacterial activity against *Escherichia coli*, *Staphylococcus aureus*, and *Candida* displayed inhibition zones of 20, 22 and 19 mm (Table 3; Fig. 3). The enhanced antibacterial efficacy of N-CDs-CS-CMC, relative to CS-CMC, is likely due to the multifaceted interactions facilitated by the N–CDs. The docking analysis revealed strong binding of N–CDs-CS-CMC with these target proteins, evidenced by exceptionally short bond lengths of approximately 2.02 Å for *E. coli* (7AB5), 1.05 Å for *S. aureus* (4QLO), and 2.73 Å for *Candida Albicans* (1YR1). These short ligand-protein bond lengths are crucial for understanding the observed antimicrobial effects, as they signify robust, stable interactions between N–CDs-CS-CMC and vital microbial components. These tight binding events, facilitated by a range of bonding mechanisms including hydrogen bonding, π-π stacking, and electrostatic interactions ^17,46,54^ are hypothesized to lead to several critical disruptions within the microbial cells. Specifically, the exceptionally short bond lengths suggest that N–CDs-CS-CMC binds deeply within the active sites or critical structural regions of these microbial proteins. This strong binding can induce conformational changes, altering the protein’s native structure and subsequently leading to the loss of its biological function. For instance, if the targeted proteins are enzymes essential for metabolic pathways or cell wall synthesis, their inactivation would directly impair bacterial viability and growth. Furthermore, while not directly shown by these specific PDBs, the strong interactions facilitated by N–CDs can also contribute to the disruption of microbial cell membranes, compromising their integrity and leading to the leakage of intracellular components. The known capacity of N-CDs to engage with nucleic acids further suggests potential interference with vital processes like DNA replication or RNA transcription ^54^. This direct correlation between the tight, short-bond interactions observed in our molecular docking simulations and the broader mechanisms of protein dysfunction, membrane disruption, and potential nucleic acid interference provides a robust explanation for the superior antimicrobial efficiency of N–CDs-CS-CMC compared to CS-CMC. The absence of such strong, short-distance interactions with microbial targets, as indicated by CS-CMC’s lack of antibacterial activity, further validates the critical role of N–CDs in facilitating these lethal interactions and ultimately leading to microbial death ^16,17,38^.

**Table 3:** Inhibition zones for CS-CMC and N–CDs-CS-CMC.

Organism	CS-CMC	*N*–CDs-CS-CMC
*Escherichia coli*	0	20 mm
*Staphylococcus aureus*	0	22 mm
*Candida albicans*	0	19 mm

### N–CDs-cellulose sulfate-carboxymethyl cellulose film as a probe for imaging Cr(VI), fungi, bacteria

**Fig. 4:**
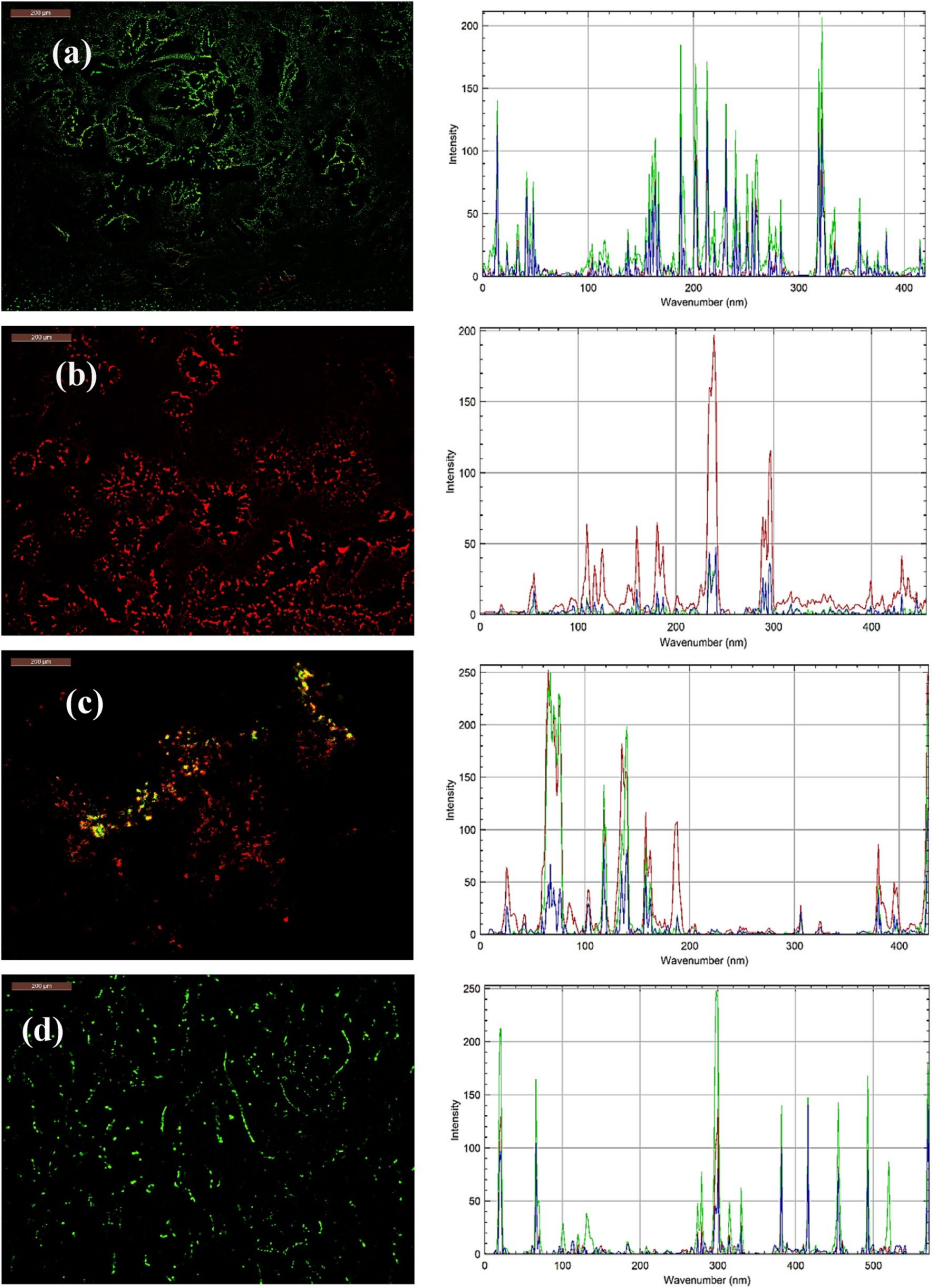

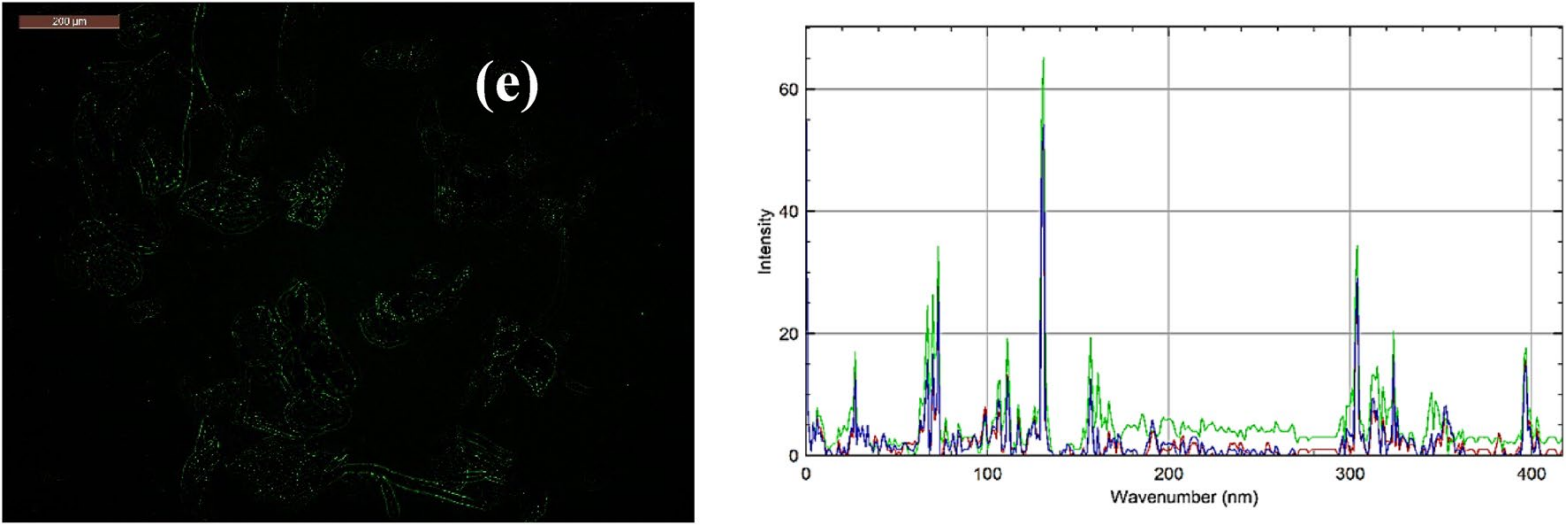
Fluorescence microscope for (**a**) N–CDs-CS-CMC before bacterial contact with plot profile fluorescence intensity, (**b**) N–CDs-CS-CMC after contact with *Escherichia* coli with plot profile fluorescence intensity, (**c**) N–CDs-CS-CMC after contact with *Staphylococcus aureus* with plot profile fluorescence intensity, (**d**) N–CDs-CS-CMC after contact with *Candida Albicans* with plot profile fluorescence intensity, and (**e**) N–CDs-CS-CMC after contact with Cr(VI) with plot profile fluorescence intensity.

The N–CDs-CS-CMC showed green fluorescence prior to bacterial exposure which proved their fluorescent properties (Fig. 4a). To evaluate the effectiveness of N–CDs-CS-CMC in fungi and bacterial detection, we selected *Escherichia coli*, *Staphylococcus aureus*, and *Candida Albicans* as our target pathogens. For confocal microscopy studies, *Escherichia coli*, *Staphylococcus aureus*, and *Candida Albicans* were harvested during the exponential growth phase. Following incubation with N–CDs-CS-CMC, the cell-conjugates were washed with double-distilled water and imaged using a fluorescence microscope. Notably, a distinct fluorescence pattern emerged upon interaction with *Escherichia coli*, *Staphylococcus aureus*, and *Candida Albicans*. In the presence of *Escherichia coli*, N–CDs-CS-CMC exhibited red fluorescence (Fig. 4b). The Gram-negative nature of *Escherichia coli* dictates the presence of a lipopolysaccharide (LPS)-rich outer membrane, a critical determinant of its environmental resilience and virulence. This outer membrane, characterized by outwardly projecting LPS molecules, effectively creates a barrier that impedes direct interactions between N–CDs and the bacterial cell wall. Consequently, this LPS-mediated steric hindrance alters the fluorescence response of the N–CDs, resulting in a modified red emission profile ^11,17^.

In the presence of *Staphylococcus aureus*, N-CDs-CS-CMC displayed a yellow-orange fluorescence (Fig. 4c). This distinct fluorescence profile arises from the higher concentration of negatively charged teichoic acids on the *Staphylococcus aureus* cell surface, relative to the LPS content in *Escherichia coli *^46^. This enhanced negative surface charge facilitates a stronger electrostatic attraction and binding of the positively charged N-CDs-CS-CMC, leading to the observed more luminous red-orange fluorescence emission. The hydrophilic nature of N-CDs-CS-CMC, conferred by oxygen, sulfur, and nitrogen functionalities, facilitates efficient internalization into *Candida albicans* cells via endocytosis (Fig. 4d). This cellular uptake is substantiated by the observed transition of the green fluorescence organization into thin, filamentous structures, indicative of successful intracellular localization ^11,46^.

The effectiveness of the N–CDs-CS-CMC film in detecting fungi and bacteria, as well as its colorimetric response to Cr(VI) (fluorescence turn off of the green color), was evaluated in this study. This colorimetric response (green to red upon Cr(VI) interaction) indicates that the N–CDs-CS-CMC film could be effectively utilized as a visual sensor for monitoring Cr(VI) levels within food packaging (Fig. 4e).

### N–CDs-cellulose sulfate-carboxymethyl cellulose film as pH-sensor for tomatoes spoilage and Cr(VI) pollution by naked eye

**Fig. 5:**
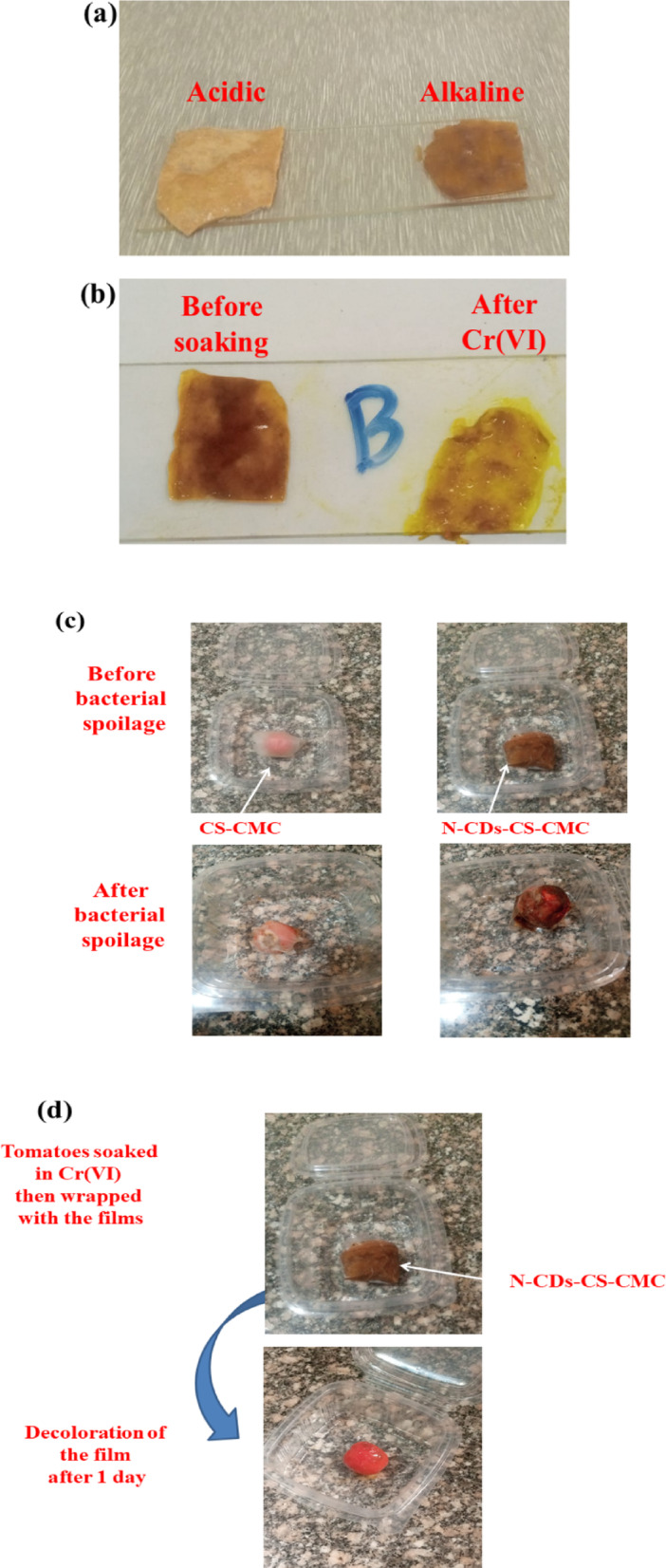
(**a**) Color response of N–CDs-CS-CMC film at acidic and alkaline media, (**b**) Color response of N–CDs-CS-CMC film at Cr(VI) with concentration: 1.85 mg/kg, (**c**) Testing of N–CDs-CS-CMC on tomatoes spoilage by bacteria, and (**d**) Testing of N–CDs-CS-CMC on tomatoes spoilage after Cr(VI) soaking.

The N-CDs-CS-CMC film, incorporating betalain-rich BR, exhibited a distinct color shift in response to varying pH conditions. Under alkaline conditions, the film displayed a yellow color, while acidic solutions induced a brown/reddish coloration (Fig. 5a). This colorimetric change is attributed to the inherent properties of betalains, the water-soluble pigments present in BR. Specifically, betacyanins, which contribute the purplish-red color, maintain their chemical structure and thus color stability within the pH range of 3 to 7. However, under alkaline conditions, the betacyanin structure undergoes transformations, leading to the disappearance of the purplish-red and the emergence of yellow, driven by the formation of betaxanthins, demonstrates that N–CDs-CS-CMC films incorporating betalains-rich BR can serve as effective pH-responsive indicators ^55^.

The decolorization of the film from its initial brown/reddish color to a bare yellow upon soaking in a chromium solution can be explained by considering the likely reactions between chromium ions and betalains which found in BR (Fig. 5b). The initial brown/reddish color is attributed to chromophores, including potentially betalains, known for their pH-sensitive color variations. When the film is immersed in the chromium solution, the chromium ions, powerful oxidizing or reducing agents, initiate chemical reactions. Chromium, particularly in its hexavalent (Cr(VI)) form, is a strong oxidant, capable of disrupting the conjugated systems within betalain molecules, which are responsible for their vibrant colors. This disruption leads to the breakdown of the chromophore’s structure, causing a loss of color. Additionally, chromium ions can form complexes with betalains, altering their electronic structure and light absorption properties. Therefore, the resulting yellow color suggests that the chromium solution effectively altered or removed the color-producing molecules, including potentially betalains, leaving behind the inherent yellow of the base material.

The native pH of tomato fruit typically ranges from 4.0 to 4.6, a factor influencing its susceptibility to microbial spoilage ^56,57^. Spoiled tomatoes exhibited a pH of 5.2 ^58^. Upon inoculation with *Escherichia coli*, *Staphylococcus aureus*, or *Candida albicans*, and subsequent application of an N–CDs-CS-CMC film with distinct pH alterations within the tomato matrix are anticipated. In the case of *Escherichia coli*, its metabolic activity is expected to contribute to a localized pH shift, potentially towards a slightly more acidic environment, as observed in other biological systems where bacterial proliferation leads to organic acid production. The *Staphylococcus aureus*, while also capable of metabolic acid production, may induce localized pH changes dependent on its specific metabolic pathways and the availability of nutrients within the tomato. The *Candida albicans*, a yeast, is known to influence pH through its fermentative processes, potentially resulting in localized pH increases or decreases based on the specific metabolic byproducts generated. The N–CDs-CS-CMC film, while primarily designed for antimicrobial activity, may also indirectly influence pH dynamics through its interaction with bacterial and fungal metabolic products. The film’s interaction with the microbes, and the tomato itself will create a complex system, and the overall pH of the tomato would be the combination of these interactions. The conversion of tomato sugars to organic acids during spoilage would also contribute to the overall pH shift. The N–CDs-CS-CMC film, by inhibiting microbial growth, aims to stabilize the pH and mitigate these spoilage-related pH changes. This resulted in tomatoes wrapped with the N–CDs-CS-CMC film taking 10 days to spoil, compared to the CS-CMC film, which led to spoilage after only 4 days. Notably, after spoilage, the N–CDs-CS-CMC film exhibited a color transformation from its initial brown/reddish color to a more intensely reddish coloration, suggesting a chemical interaction between the film’s components and the byproducts of microbial degradation (Fig. 5c; Table 4). In contrast, when Chromium polluted tomatoes were wrapped with the N–CDs-CS-CMC film, a distinct phenomenon was observed. The film underwent rapid decolorization, transitioning from its initial brown/reddish color to a bare yellow within a single day (Fig. 5d). This decolorization coincided with a noticeable increase in the tomato’s translucency, effectively rendering the red color of the tomato fruit more transparent. This suggests a direct interaction between the chromium within the film and the tomatoes, leading to both the film’s color change. This rapid decolorization of the film acts as a direct indicator of the presence of Cr(VI) in the tomatoes. The underlying mechanism, as discussed previously, likely involves the interaction of Cr(VI) ions (a strong oxidant) with the betalain chromophores present in the film. This interaction could lead to the breakdown of these color-producing molecules or the formation of new, colorless or yellow-colored complexes. Furthermore, an equally significant observation was the accompanying increase in the tomato’s translucency, making its natural red color appear more transparent. This suggests a broader interaction than just the film sensing the chromium. It implies a direct exchange or effect between the Cr(VI) within the tomato and the components of the film. This makes the N–CDs-CS-CMC film a promising tool for rapid and observable detection of chromium pollution in fresh produce.

**Table 4:** Shelf life of tomatoes by using CS-CMC and N–CDs-CS-CMC.

Film	Shelf life (days)
CS-CMC	4
N–CDs-CS-CMC	10

### Beyond plastic: the Eco-Conscious degradation of CS-CMC and N–CDs-CS-CMC films

A key environmental benefit of both the CS-CMC and N–CDs-CS-CMC films is their exceptional biodegradability, a crucial factor considering the global concern over plastic pollution. Unlike traditional petroleum-based plastic packaging, which can persist in landfills and natural environments for hundreds of years, these films are derived from natural biopolymers and are designed to seamlessly integrate back into the ecological cycle. Crucially, preliminary assessments have demonstrated a remarkable degradation rate for these films in soil. Specifically, both the plain CS-CMC film and the more functionalized N–CDs-CS-CMC film showed significant breakdown and even complete disappearance within a single day when buried in soil. This rapid decomposition highlights their eco-friendly nature and their potential to mitigate the environmental impact associated with food packaging and sensing applications. This rapid biodegradability is largely attributed to the microbial activity within the soil. Soil microorganisms, including various bacteria and fungi, possess the enzymatic machinery necessary to break down complex biopolymers like CS and CMC into simpler, non-toxic components, such as water, carbon dioxide, and biomass. The quick degradation observed ensures that these films do not contribute to long-term waste accumulation, offering a sustainable alternative that aligns with principles of a circular economy.

## Conclusions

This study successfully developed and characterized an N–CDs-CS-CMC composite film, demonstrating its potential as a multifunctional material for tomato packaging and sensing applications. DFT calculations revealed a significant enhancement in polarity and reduced energy gap in the N–CDs-CS-CMC composite compared to CS-CMC, indicating improved molecular interactions and enhanced charge transfer capabilities. FTIR and SEM analyses further confirmed the successful incorporation of N–CDs into the CS-CMC matrix, leading to a film with smaller, more uniform pores and increased surface area, crucial for efficient sensing and controlled diffusion. The N–CDs-CS-CMC film exhibited potent antimicrobial activity against *Escherichia coli*, *Staphylococcus aureus*, and *Candida albicans*, attributed to the multifaceted interactions between N–CDs and bacterial components, as supported by molecular docking studies. The film’s ability to detect these pathogens through distinct fluorescence patterns underscores its potential for real-time monitoring of microbial contamination. Furthermore, the incorporation of BR within N–CDs-CS-CMC endowed the film with pH-responsive colorimetric properties, enabling naked-eye detection of spoilage-related pH changes in tomatoes. The N–CDs-CS-CMC’s ability to delay tomato spoilage by extending shelf life to 10 days, compared to 4 days for the CS-CMC film, highlights its efficacy in preserving food quality. Moreover, the distinct color changes observed in the film upon exposure to chromium-polluted tomatoes, transitioning from brown/reddish to yellow, coupled with increased tomato translucency, showcases its potential for detecting heavy metal contamination. Several challenges and limitations were identified during this study. One significant area for further development lies in the long-term stability of the film’s sensing performance, particularly under varying environmental conditions such as prolonged exposure to humidity, UV light, or extreme temperatures, which could potentially impact the integrity of the N–CDs or the betalain chromophores. Finally, although preliminary biodegradability was promising, a more exhaustive study on the biodegradation kinetics and end-products under diverse environmental conditions (e.g., different soil types, aquatic environments) would provide a more complete environmental impact assessment. Addressing these limitations in future work will be essential for the widespread adoption and commercialization of this novel sensing technology.

## Data Availability

Data is provided within the manuscript.
